# Electrochemical Deposition of Silver Nanoparticle Assemblies on Carbon Ultramicroelectrode Arrays

**DOI:** 10.1002/cphc.202400791

**Published:** 2025-01-08

**Authors:** Courtney J. Weber, Natalie E. Strom, Emma M. Vagnoni, Olja Simoska

**Affiliations:** ^1^ Department of Chemistry and Biochemistry University of South Carolina 631 Sumter Street Columbia, SC 29208 United States

**Keywords:** Carbon ultramicroelectrode arrays, Electrocatalysis, Electrochemistry, Silver nanoparticles, Electrodeposition

## Abstract

Silver nanoparticle (AgNP) assemblies combined with electrode surfaces have a myriad of applications in electrochemical energy storage and conversion devices, (bio)sensor development, and electrocatalysis. Among various nanoparticle synthesis methods, electrochemical deposition is advantageous due to its ability to control experimental parameters, enabling the formation of low‐nanoscale (<50 nm) particles with narrow size distributions. Herein, we report the electrodeposition of AgNPs on a unique electrode platform based on carbon ultramicroelectrode arrays (CUAs), exploring several experimental variables including potential, time, and silver ion concentration. Extensive scanning electron microscopy analysis revealed that more reductive deposition potentials resulted in higher counts of smaller‐sized AgNPs. While previous studies have employed planar, macro‐sized electrodes with millimolar silver ion concentrations and minute‐long times for AgNP electrodeposition, our results demonstrate that lower Ag^+^ concentrations (50–100 μM) and shorter deposition times (15–30 s) are sufficient for successful AgNP formation on CUAs. These findings are attributed to enhanced mass transfer from the radial diffusion of the array‐based CUAs. The quantity of deposited Ag was determined to be 1100±200 nmol cm^−2^, consistent with AgNP‐modified CUA electrocatalytic activity for hydrogen peroxide reduction. This study emphasizes the importance of carefully considering AgNP electrodeposition parameters on unconventional electrode surfaces.

## Graphical abstract

Silver nanoparticle (AgNP) electrochemical deposition on carbon ultramicroelectrode arrays (CUAs) with varying deposition potentials, deposition times, and silver ion concentrations to achieve sufficient particle dispersion, size, and count. The AgNP‐modified CUAs were characterized by determining the deposited Ag quantity and demonstrating enhanced electrocatalytic activity for the hydrogen peroxide reduction.

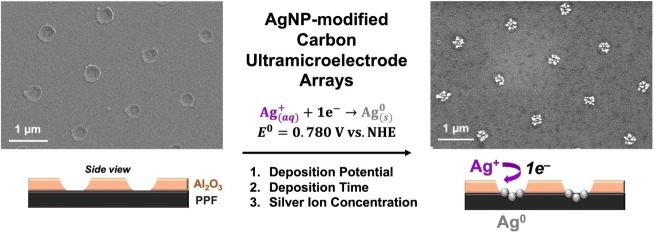

## Introduction

Silver nanoparticle (AgNP) assemblies are nano‐sized materials that can be employed in several scientific applications, including (bio)electrochemical sensing, energy storage and conversion, electrocatalysis, environmental remediation, and pharmaceuticals.[^1^, ^2^, ^3^, ^4^, ^5^, ^6^, ^7^, ^8^, ^9^, ^10^, ^11^, ^12^, ^13^, ^14^] Specific applications of AgNP assemblies encompass the development of better‐performing fuel cells,^[7]^ drug delivery systems,^[15]^ bacterial infection therapy,[^16^, ^17^, ^18^, ^19^, ^20^, ^21^] wastewater treatment,^[8]^ coatings for medical devices,^[6]^ cosmetics,^[9]^ wound dressings,^[2]^ as well as sensor development for the detection of environmental contaminants[^10^, ^11^] and health biomarkers.[^12^, ^13^, ^22^] The widespread utility of AgNPs is attributed to their unique physical and chemical properties. Due to their high surface area and nanoscale dimensions, AgNPs exhibit unique optical behavior,[^11^, ^23^] enhanced electrical conductivity and electrocatalytic activity,[^6^, ^24^, ^25^] improved biocompatibility,[^17^, ^26^, ^27^] and antimicrobial properties[^16^, ^17^, ^18^, ^19^, ^20^, ^21^] in comparison to solid bulk silver. Furthermore, the enhanced electrocatalytic properties of AgNPs can be harnessed by modifying electrode surfaces with these materials.[^14^, ^28^, ^29^]

To achieve the desirable chemical and physical properties of AgNP‐modified electrodes for a wide range of electrochemical applications, several methods of AgNP fabrication have been examined previously.[^6^, ^18^, ^24^, ^30^] AgNPs can be synthesized via physical, chemical, biological, photochemical, or electrochemical processes. Physical AgNP synthesis includes vapor condensation or laser ablation steps, both of which require bulk silver metal, high energy input, and prolonged formation time.[^31^, ^32^] The photochemical fabrication of AgNPs is a more simplistic method that employs aqueous silver cations, however, it is associated with longer synthesis times and forms less desirable, larger particles with broad size distributions.[^33^, ^34^] In chemical synthesis, chemical reducing agents are utilized to consistently form small‐sized AgNPs in a colloidal solution, but this approach often requires the use of harsh chemicals and reaction conditions.[^35^, ^36^, ^37^] The biological fabrication of AgNPs employs naturally occurring reducing agents from plants and microorganisms to form colloidal particles. However, this method is difficult to reproduce at a larger scale and the reaction is often slow and inefficient.[^38^, ^39^] To address the challenges of the aforementioned methods, recent studies have utilized electrochemical deposition of AgNPs on electrode surfaces as a promising alternative.[^4^, ^5^, ^10^, ^11^, ^12^, ^13^, ^16^, ^22^, ^40^] This technique yields consistent particle formation without the use of various reducing reagents and costly materials, harsh reaction conditions, high energy input, prolonged synthesis times, and scale‐up limitations.[^1^, ^41^] In addition, electrochemical methods enable control of particle size, dispersion, and morphology by modifying deposition parameters, such as applied potential, time, metal ion concentration, and supporting electrolyte composition.[^42^, ^43^]

Previous studies have evaluated the impact of electrodeposition parameters on AgNP formation, electrochemical behavior, and electrocatalytic performance on various electrode materials, including glassy carbon,[^1^, ^4^, ^10^, ^29^] graphite,^[11]^ multi‐walled carbon nanotubes,[^12^, ^13^] nitrogen‐containing mesoporous carbon,^[5]^ boron‐doped diamond,^[14]^ gold,^[16]^ and indium tin oxide (ITO) electrodes.[^22^, ^40^] The Zamborini Research Group evaluated the impact of electrochemical potential and deposition time on the formation of Ag nanomaterials on ITO electrodes. This study demonstrated that more reductive potentials resulted in a higher density of Ag assemblies and longer deposition times formed larger Ag amounts on the electrode surface.^[44]^ Ustarroz and co‐workers reported the electrodeposition of AgNPs on carbon‐coated gold transmission electron microscopy (TEM) grids to improve nanoparticle structural analysis capabilities. They found that shorter deposition times generated higher counts of smaller‐sized particles with a narrower size distribution when compared to longer time regimes.^[1]^ Ajermoun et al. studied the electrocatalytic behavior of AgNP‐modified graphite electrodes toward insecticide detection in food and beverage applications. The best electrocatalytic activity (highest electrical current response) was achieved from the electrodes with AgNPs deposited at shorter times, more reductive potentials, and lower silver ion concentrations.^[11]^ The Unwin Research Group demonstrated the effects of AgNP formation and size on the inherent antimicrobial behavior of these materials using scanning electrochemical cell microscopy.^[16]^ AgNP‐modified electrode samples associated with lower Ag^+^ concentrations and shorter deposition periods achieved particle sizes of less than 10 nm, which displayed greater antimicrobial activity when tested in live cell cultures.^[16]^ These research works demonstrate the importance of establishing electrodeposition parameters to achieve optimal AgNP formation for desired applications.

While previous studies have advanced the understanding of AgNP deposition on various electrode materials, the exploration of micro‐ and nano‐electrodes in an array‐based geometry remains limited. To address this gap, we examined and characterized the electrodeposition of AgNPs on a unique array‐based nanoelectrode platform based on carbon ultramicroelectrode arrays (CUAs). The carbon material used in the CUA design is pyrolyzed photoresist film (PPF), which is conductive, hard, and chemically inert with electrochemical behavior similar to glassy carbon.^[45]^ The CUA platform consists of approximately 3×10^9^ individual carbon ultramicroelectrodes (~90 nm in radius) per electrode area (cm^2^) organized in an array‐based fashion. Surrounding the individual carbon ultramicroelectrodes in the array is a 10 nm insulating aluminum oxide (Al_2_O_3_) layer, which acts as a dielectric to store and organize charge. As such, the Al_2_O_3_ layer greatly diminishes the noise associated with double‐layer charging, resulting in improved signal‐to‐noise (S/N) ratios compared to planar electrodes made entirely of PPF (i. e., Macro electrodes).[^46^, ^47^] The distinctive combination of the CUA electrode materials, array‐based geometry, and nano‐scale electrode sizes present several advantages in electrochemical applications, including limited surface adsorption, fast electrode response times, and sensitive analyte detection in complex biological matrices.[^48^, ^49^, ^50^, ^51^, ^52^, ^53^] The facile and highly reproducible CUA fabrication procedure has been previously established and well‐characterized to ensure consistent, enhanced electrochemical performance.[^46^, ^47^, ^48^, ^49^, ^50^, ^51^, ^52^, ^53^] Additionally, the photoresist material that constitutes the PPF can be diluted to produce optically transparent electrodes for spectroelectrochemical applications.^[49]^ Finally, the CUA electrodes can be easily tailored and modified with nanomaterials for applications in electrochemical (bio)sensing, electrocatalysis, and the development of batteries and fuel cells.[^48^, ^49^, ^50^, ^51^, ^52^, ^53^, ^54^]

Herein, we report a detailed characterization and analysis of AgNP electrodeposition on CUAs. The redox behavior of silver at the CUA surface was evaluated using cyclic voltammetry (CV) to determine the experimental AgNP deposition potentials for desired AgNP formation. Amperometry was utilized for the electrodeposition of AgNPs on CUAs at various combinations of deposition potentials, times, and silver nitrate (AgNO_3_) concentrations. The amount of silver on AgNP‐modified electrode samples was determined from the amperometric data to evaluate the impact of specified experimental parameters on AgNP deposition. Particle dispersion, morphology, size, and nanoparticle count data were achieved through extensive scanning electron microscopy (SEM) characterization. The enhanced electrocatalytic activity of AgNP‐CUAs was demonstrated in the electrochemical reduction of hydrogen peroxide (H_2_O_2_). Overall, our results show the influence of electrodeposition parameters, including applied potential, time, and Ag^+^ concentration, on the formation of AgNPs on CUAs. Compared to previous AgNP electrodeposition reports on planar, macro‐sized electrodes,[^1^, ^4^, ^5^, ^10^, ^11^, ^12^, ^13^, ^22^, ^29^, ^55^] we achieved optimal AgNP formation using lower, micromolar concentrations and shorter deposition times due to unique CUA geometry, electrode sizes, and associated radial diffusion profiles. This study demonstrates that our novel array‐based CUA electrode platform enables effective electrochemical deposition of AgNPs with uniform sizes. These nanoparticles have a wide range of applications, including electrocatalysis, energy conversion and storage, and electroanalytical detection.

## Experimental

### Chemicals and Materials

All materials and chemicals were used as received. Quartz microscopic slides with an area of 6.45 cm^2^ and a thickness of 1 mm, used for electrode fabrication, were purchased from Technical Glass Productions. Polystyrene spheres (Polybead ®) with a diameter of 1.54 μm were acquired from Polysciences, Inc. Photoresist AZ1518 from Integrated Micro Materials (iMicromaterials). Silver nitrate (AgNO_3_) and hydrogen peroxide solution (H_2_O_2_, 30 wt. % in H_2_O) were purchased from Sigma‐Aldrich. Potassium nitrate (KNO_3_) was purchased from Fischer Scientific. Sodium phosphate buffer was utilized, containing sodium phosphate monobasic (H_2_NaO_4_P) and sodium phosphate dibasic (HNa_2_O_4_P), obtained from Sigma‐Aldrich, as well as potassium chloride (KCl) obtained from Fisher Scientific. Water utilized for all experiments was dispensed from a Millipore Sigma Milli‐Q® EQ 7000 ultrapure water purification system with a resistivity of 18.2 MΩ⋅cm at 25 °C.

### Procedure Steps for Fabrication of Carbon Ultramicroelectrode Arrays (CUAs)

The CUA fabrication was performed using previous procedures reported elsewhere.[^46^, ^47^, ^48^, ^49^, ^50^, ^51^, ^52^, ^53^] In brief, the quartz microscopic slides were first thoroughly cleaned in a piranha solution (3 : 1 H_2_SO_4_:30 % H_2_O_2_) for the removal of contamination from organic compounds. The slides were then spin coated at 600 rpm for 1 min (WS‐650Mz‐23NPPB spin coater, Laurell Technologies) with undiluted AZ1518 photoresist. The applied photoresist film was pyrolyzed following a tube furnace‐controlled heating procedure[^46^, ^47^] to form pyrolyzed photoresist film (PPF). The tube furnace atmosphere was first purged for 15 s with forming gas (5 % H_2_:95 % N_2_) at an approximate flow rate of 100 mL min^−1^. The photoresist‐covered slides were then heated to 1000 °C at a rate of 5 °C min^−1^. This temperature was held for 1 h, and the system was cooled to room temperature at the same rate, yielding planar, pyrolyzed photoresist film (PPF)‐based Macro electrodes (i. e., carbon electrodes without the array). The PPF slides were stored for at least 3 days in an oxygenated environment to allow for the stabilization of the oxide layers.^[56]^ Previous reports of PPF prepared by this method indicate film thickness of 250±20 nm, roughness of 0.39±0.07 nm, and sheet resistance of 97±3 Ω/sq. (ohms per square).^[45]^ After pyrolysis, polystyrene spheres (PSSs) with 1.54 μm diameter were drop cast onto the PPF. Atomic layer deposition (ALD) (AT410 bench‐top ALD system, Anric Technologies) was used to deposit a 10 nm‐layer of aluminum oxide (Al_2_O_3_). Following ALD, sonication steps were performed in methanol, acetone, ethanol, and water to remove the PSSs, forming an ordered array of individual carbon ultramicroelectrodes at the sites where the spheres contacted the PPF. Lastly, a drying step was performed with N_2_ gas prior to experimentation.

### Silver Nanoparticle (AgNP) Electrochemical Deposition and Characterization

All electrochemical experiments utilized a three‐electrode cell setup with either Macro electrodes or CUAs as the working electrode (WE), a saturated calomel electrode (SCE) as the reference electrode (RE), and platinum mesh (Pt) as the counter electrode (CE). All electrochemical experiments were conducted on CH650E, CH660E, and CH440C potentiostats (CH Instruments). Single‐potential‐step chronoamperometry was employed in the electrochemical deposition of silver nanoparticles (AgNPs) on CUAs by applying a constant reductive potential to reduce Ag^+^ cations in solution to solid Ag at the surface of the WE. The Ag^+^ source was silver nitrate (AgNO_3_) at deposition concentrations of 50, 100, and 250 μM. All silver deposition solutions also contained 0.5 M of potassium nitrate (KNO_3_) as the supporting electrolyte. The potential was held at an initial value of 0.2 V vs. SCE for 1 s, then stepped to the associated electrodeposition potential (−0.3, −0.4, −0.5, or −0.6 V vs. SCE) and held for 15, 30, 60, or 120 s. The sensitivity was 0.001 A V^−1^ with a sample interval of 0.001 s. Cyclic voltammetric (CV) data for the characterization of 250 μM AgNO_3_ in 0.5 M KNO_3_ solution on CUAs was performed in a potential window of −0.6 to 0.8 V vs. SCE at a scan rate of 0.1 V s^−1^, with a sample interval of 0.001 V, quiet time of 2 s, and sensitivity of 0.001 A V^−1^. CV measurements for the hydrogen peroxide (H_2_O_2_) reduction study employed a solution of 10 mM H_2_O_2_ in 0.1 M sodium phosphate buffer (SPB) in a potential window of −0.9 to −0.2 V vs. SCE, at a scan rate of 0.05 V s^−1^, with a sample interval of 0.001 V, a quiet time of 2 s, and a sensitivity of 0.001 A V^−1^. Excel and OriginLab Graphing and Analysis Software were utilized to plot and analyze experimental data. All electrochemical results were presented following the classic polarographic convention, where the x‐axis displays oxidative (more positive) potentials on the left and reductive (more negative) potentials on the right. Correspondingly, the y‐axis shows anodic currents as negative values on the bottom and cathodic currents as positive values on the top.

### Scanning Electron Microscopy (SEM) Sample Preparation and Imaging

Following electrochemical deposition, AgNP‐modified CUA electrodes were rinsed with MilliQ ultrapure water and dried with N_2_ to prepare the samples for SEM analysis. SEM and energy‐dispersive X‐ray spectroscopy (EDX) were conducted in the Electron Microscopy Center, a shared facility at the University of South Carolina. For AgNP imaging experiments, a Zeiss Gemini500 FESEM model was used. The procured SEM images were further analyzed with ImageJ software to perform AgNP size measurements. For the SEM analysis, each image was imported into ImageJ software where the scale was set based on a known scale bar in nanometers. The area measurement tool was employed to calculate the particle squares. For nanoparticles with irregular shapes, Feret's diameter was utilized to obtain an accurate measurement of their diameter. Each SEM image underwent contrast enhancement and thresholding to improve visibility and ensure nanoparticles were properly highlighted. After these adjustments in ImageJ, the nanoparticle analyses were conducted.

## Results and Discussion

### Fundamental Voltammetric Study of Silver Redox Behavior on Carbon Ultramicroelectrode Arrays (CUAs)

Previous studies have evaluated the redox behavior of silver cation (Ag^+^) solution at electrode surfaces for the electrodeposition of silver nanoparticles (AgNPs).[^1^, ^4^, ^13^, ^16^, ^22^, ^57^, ^58^] The single‐electron redox mechanism for silver reduction and the associated standard reduction potential are displayed in Equation (1). Upon the application of reductive potential to the working electrode, with respect to the reference electrode, Ag^+^ ions in solution gain an electron and are electrochemically reduced to form solid Ag at the electrode surface, resulting in AgNPs. The formation of nanoparticles, rather than a metal film on the electrode surface, can be attributed to the thermodynamic favorability of deposition on various substrate materials. After the initial Ag nucleation sites are formed on the electrode surface, subsequent silver deposition occurs at the preexisting Ag sites rather than on the electrode substrate material. This process is supported by the Volmer‐Weber growth mechanism, resulting in the formation of three‐dimensional silver aggregates, or nanoparticles, on the electrode surface, instead of thin silver films.[^22^, ^57^, ^58^, ^59^, ^60^, ^61^]

(1) $${Ag}_{\left(aq\right)}^{+}+{1e}^{-}\to {Ag}_{\left(s\right)}^{0}\;\;E^{0}=0.780\;V\;vs.\;NHE$$

**Figure 1:**
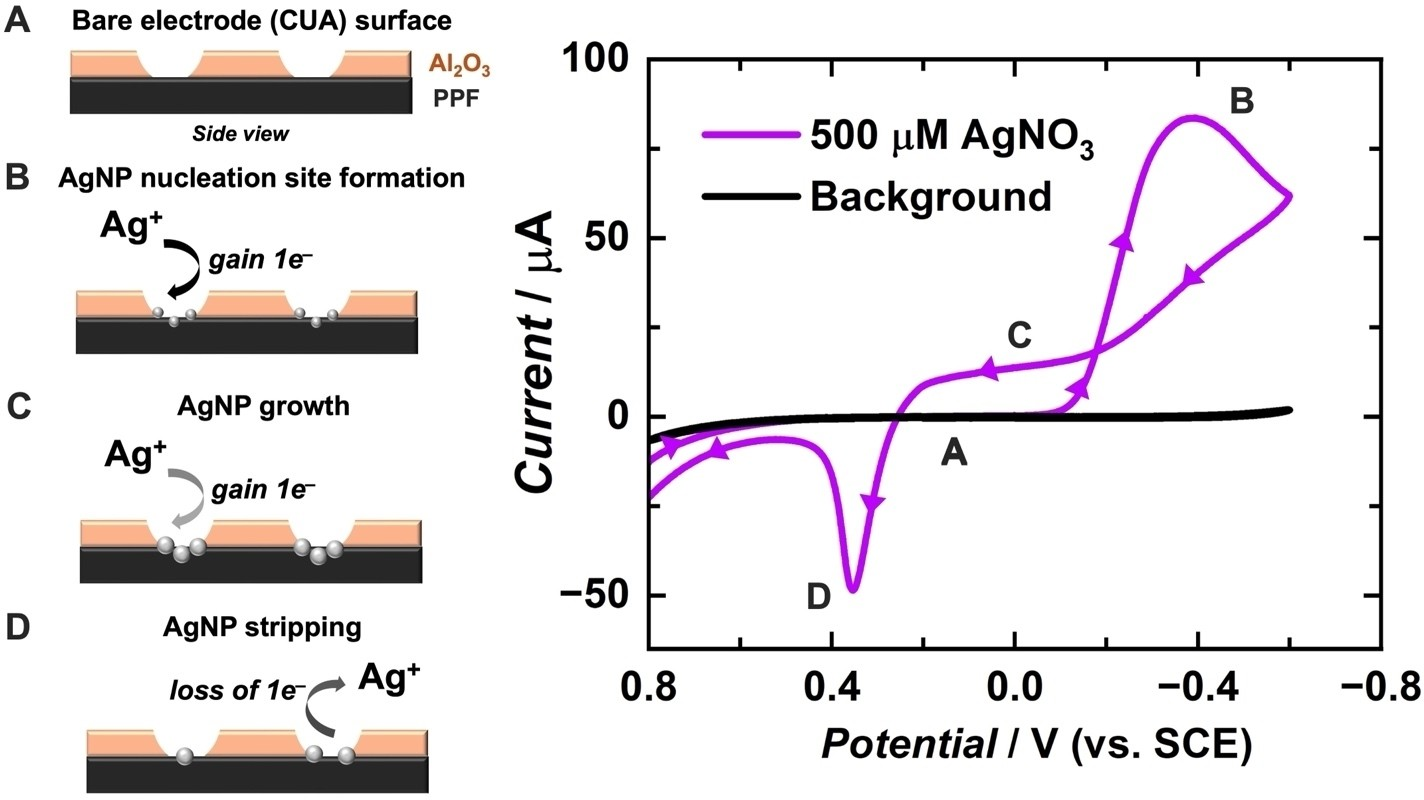
Graphical representation of silver nanoparticle (AgNP) nucleation, growth, and stripping mechanisms (A–D). These mechanisms occur over a wide potential range, as displayed in the current response of the cyclic voltammogram (CV, right) of 500 μM AgNO_3_ solution with 0.5 M KNO_3_ supporting electrolyte at a scan rate of 100 mV s^−1^. The potential was swept from an initial value of 0.2 V, then to −0.6 V, followed by 0.8 V, then back to the initial 0.2 V value. All potentials are reported versus the saturated calomel reference electrode (SCE).

Figure 1 shows cyclic voltammetric data of AgNO_3_ solution (source of Ag^+^) obtained with carbon ultramicroelectrode arrays (CUAs) in an electrochemical potential window ranging from 0.8 to −0.6 V vs. SCE. The diffusion‐controlled electrical current response associated with the rate of silver electron transfer is plotted with respect to applied electrochemical potential (i. e., driving force).[^62^, ^63^] Figure 1A–D graphically represents the electrochemical processes associated with AgNP deposition on CUAs for each specified region in the cyclic voltammogram. The CV current‐potential trace was initiated at 0.2 V vs. SCE, where a negligible faradaic current signal was observed (Figure 1A). The first potential sweep to −0.6 V vs. SCE resulted in a cathodic current response, indicating the electrochemical reduction of Ag^+^ at the CUA surface to initiate AgNP nucleation and growth (Figure 1B). In general, higher AgNO_3_ concentrations yield greater electrical current signal due to the greater availability of Ag^+^ species in solution. This trend is demonstrated with AgNO_3_ in Figure S1A. In the reverse, positive potential sweep from −0.6 V to 0.8 V vs. SCE, further AgNP growth occurs in the crossover region from approximately −0.2 V to 0.2 V vs. SCE, represented by the electrical current responses in Figure 1C that were not observed in the first negative‐direction sweep. This region of current‐potential trace crossover has been previously reported as the “nucleation loop,” which is indicative of the aforementioned Volmer‐Weber growth mechanism for electrodeposition of 3D particles.[^1^, ^4^, ^22^, ^57^, ^58^] This growth event can also be observed in multiple, subsequent CV scans (Figure S1B). A higher current signal is shown in the nucleation loop region on the second scan than on the first scan, confirming that less driving force (lower reductive electrochemical potential) is necessary to initiate silver deposition on preexisting AgNP assemblies than on the carbon electrode surface.[^4^, ^22^, ^57^, ^58^] As the potential is swept in the positive direction, the characteristic anodic stripping peak is observed for the oxidation of silver (Figure 1D).^1^ The cyclic voltammetric results in Figure 1 establish the electrochemical behavior of silver on CUAs, which is consistent with previous reports on planar electrode surfaces.[^1^, ^4^, ^13^, ^16^, ^22^, ^58^]

### Evaluation of Electrodeposition Parameters on Silver Nanoparticle (AgNP) Formation on Carbon Ultramicroelectrode Arrays (CUAs)

**Figure 2:**
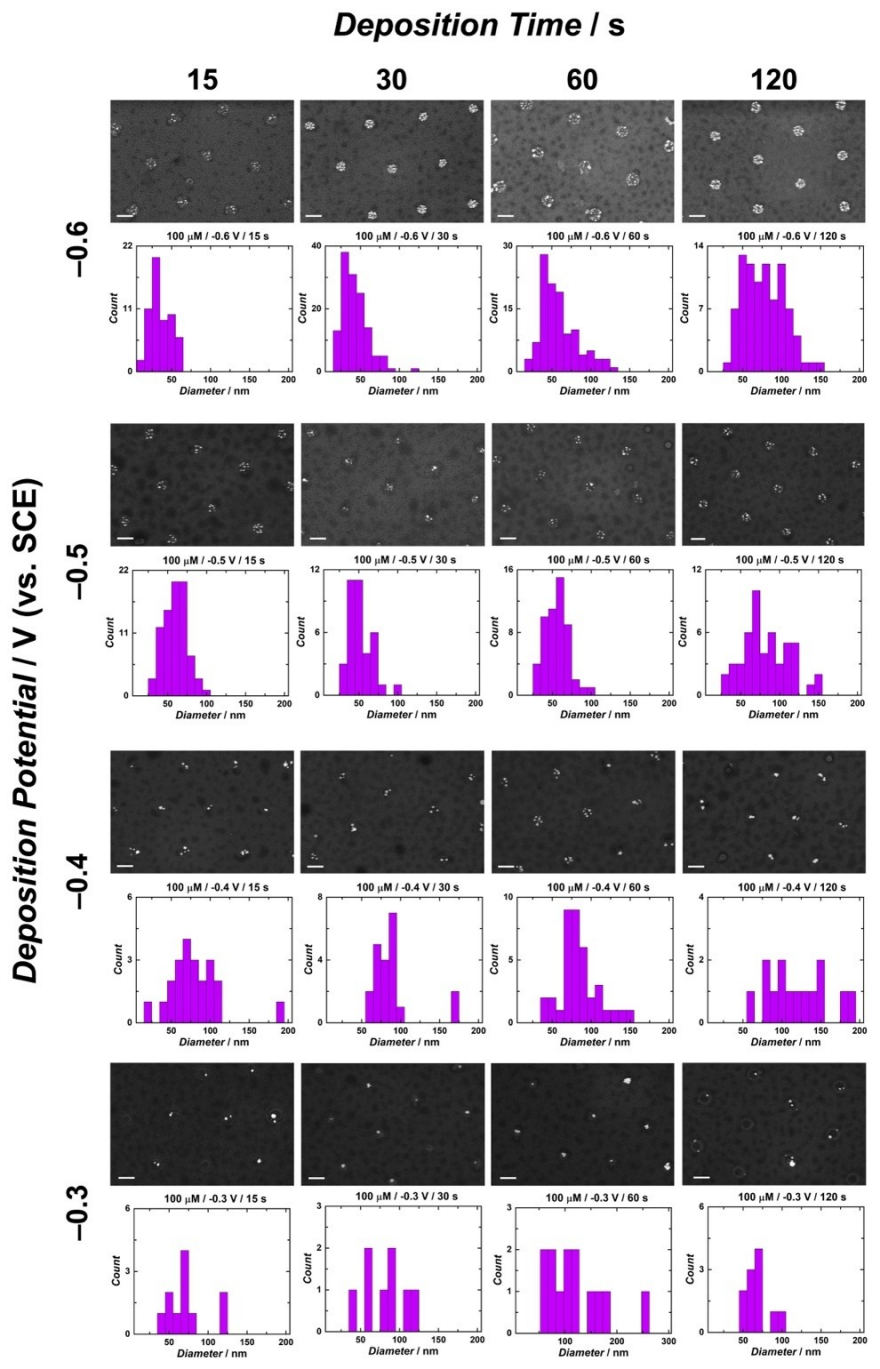
Silver nanoparticle (AgNP) size distribution histograms displayed with representative scanning electron microscopy (SEM) images for each electrodeposition potential and time parameters using 100 μM AgNO_3_ in 0.5 M KNO_3_ supporting electrolyte solution on carbon ultramicroelectrode arrays (CUAs). The data is organized according to deposition time (increasing from left to right) and potential (less reductive, or more positive, from top to bottom). Measurements for particle diameters, as displayed in the histograms, were recorded from *n*=9 individual electrodes of the same area on one CUA surface. The scale bars in each SEM micrograph are 500 nm in length.

**Figure 3:**
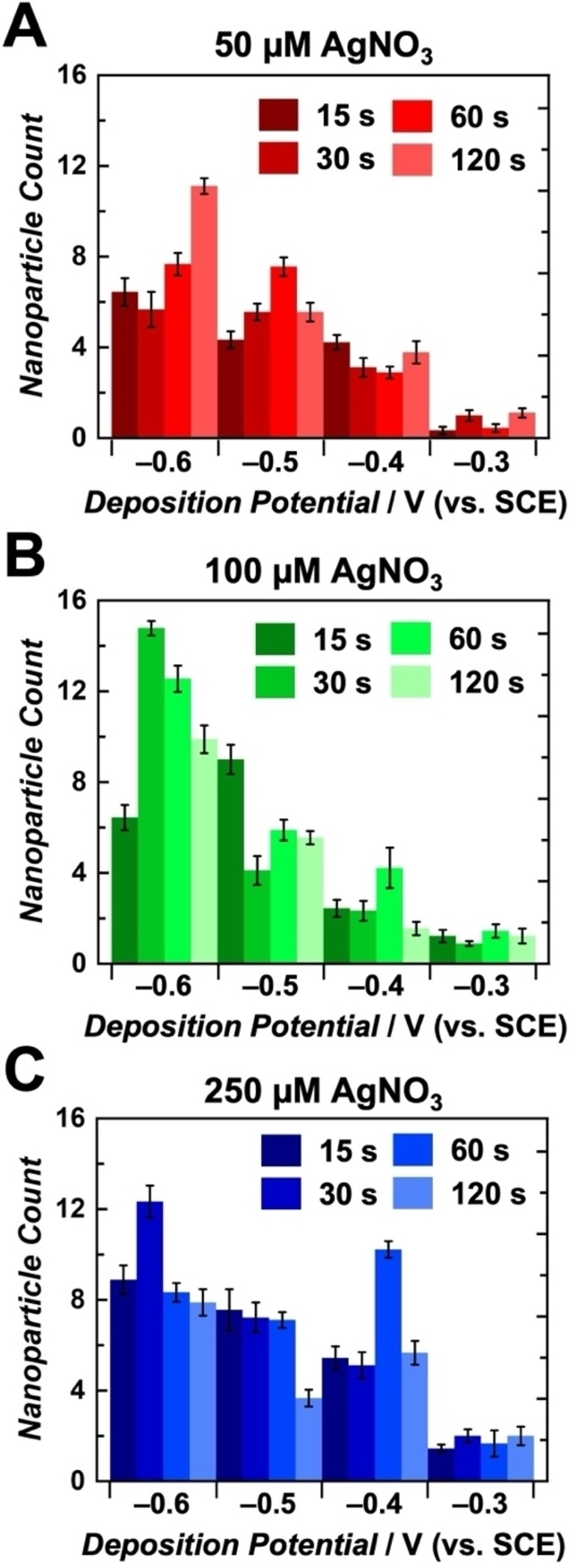
Silver nanoparticles (AgNPs) were counted on *n*=9 individual electrodes on a single carbon ultramicroelectrode array (CUA) platform at each associated deposition parameter. Plots are organized in order of increasing silver ion concentration, namely (A) 50 μM AgNO_3_, (B) 100 μM AgNO_3_, and (C) 250 μM AgNO_3_. Deposition times are portrayed by color gradients, with darker colors indicating shorter times and vice versa, and these are grouped by deposition potential displayed on the *x*‐axis.

Electrochemical methods of nanoparticle deposition are advantageous for controlling particle formation, specifically in terms of size, count, morphology, and dispersion across an electrode surface. This control is achieved through the adjustment of experimental parameters during the electrodeposition process. Amperometry is often utilized in nanoparticle electrodeposition due to the direct control over applied potential and time.[^1^, ^4^, ^5^, ^10^, ^11^, ^12^, ^13^, ^22^, ^29^, ^55^] This method applies a constant potential and measures the electrical current response, correlating to the rate of electron transfer processes, as a function of time.^[63]^ In this study, single‐potential‐step chronoamperometry was used for the electrodeposition of AgNPs on CUAs to enable precise control of deposition potential and time, while also varying silver ion concentration. Multiple values of each parameter were evaluated for the preparation of AgNP‐modified CUAs, specifically (1) deposition potentials of −0.3, −0.4, −0.5, and −0.6 V vs. SCE, (2) deposition times of 15, 30, 60, and 120 s, and (3) AgNO_3_ concentrations of 50, 100, and 250 μM. Energy dispersive x‐ray spectroscopy (EDX) was employed to confirm silver formation on the electrode surfaces (Figure S2). Scanning electron microscopy (SEM) was then employed for the extensive characterization of AgNP‐CUA samples modified with various deposition parameters. Figures 2, S3, and S4 display SEM images and histograms with particle measurement counts of the AgNP‐CUA samples at all deposition potentials and times for silver ion concentrations of 50, 100, and 250 μM, respectively. The particle size histograms show AgNP diameter measurements from nine individual nanoscale electrodes (*n*=9) within the CUA. Particle counts for the same nine replicates within each AgNP‐CUA sample are displayed in Figure 3. The total number of AgNPs on a CUA electrode surface can be estimated by multiplying the particle counts by the total number of ultramicroelectrodes in the arrays, which consists of approximately 3×10^9^ ultramicroelectrodes cm^−2^ as determined from SEM evaluations.

Particle size and AgNP count data from Figures 2, 3 S3, and S4 reveal clear trends in the effects of deposition potential, time, and Ag^+^ concentrations on the formation of AgNPs on CUAs. Specifically, in terms of applied deposition potential, SEM data demonstrate a distinct relationship between the applied potential as the electrochemical driving force and AgNP formation under all deposition times and silver ion concentration conditions. The results indicate that more reductive deposition potentials yield higher counts of smaller‐sized particles, while less reductive potentials result in fewer, larger particles. These findings align with previous studies and can be explained by understanding the effects of the applied electrochemical driving force on nanoparticle nucleation.[^4^, ^11^, ^22^, ^58^] When a higher driving force (i. e., more reductive potential (e. g., −0.6 V vs. SCE)) is applied to the working electrode, nanoparticle deposition becomes more favorable, leading to the formation of more AgNP nucleation sites on the CUA surface. This increased AgNP nucleation results in reduced particle growth, producing a larger number of smaller‐sized AgNPs on the CUAs. Conversely, at less reductive deposition potentials (e. g., −0.3 V vs. SCE), associated with a lower electrochemical driving force, fewer nucleation sites are formed, leading to the growth of larger AgNPs. Overall, these observed trends in the influence of the electrochemical deposition potential on nanoparticle size and count are consistent with previous reports.[^1^, ^11^, ^16^, ^44^]

In addition to AgNP trends at different deposition potentials, our results demonstrate that extended deposition times (120 s) and higher Ag^+^ concentrations (250 μM) consistently lead to the formation of larger particles at all deposition potentials. Longer deposition periods allow more time for particle growth, resulting in larger AgNPs, which is consistent with previously reported trends in electrodeposition.[^1^, ^11^, ^16^, ^44^] Similarly, higher AgNO_3_ concentrations promote the formation of larger AgNPs due to the increased availability of Ag^+^ at the CUA electrode‐solution interface. Based on SEM analysis (Figure 3), the optimal electrodeposition values for AgNP deposition on CUAs are a deposition potential of −0.6 V vs. SCE, a deposition time of 30 s, and an AgNO_3_ concentration of 100 μM. Notably, this study achieves successful AgNP formation with second‐long deposition times and micromolar Ag^+^ concentrations, whereas previous studies using macrometer‐sized planar electrodes have reported deposition times of several minutes and silver ion concentrations in the millimolar range to produce comparable results.[^1^, ^5^, ^11^, ^12^, ^13^, ^22^, ^29^, ^55^]

**Figure 4:**
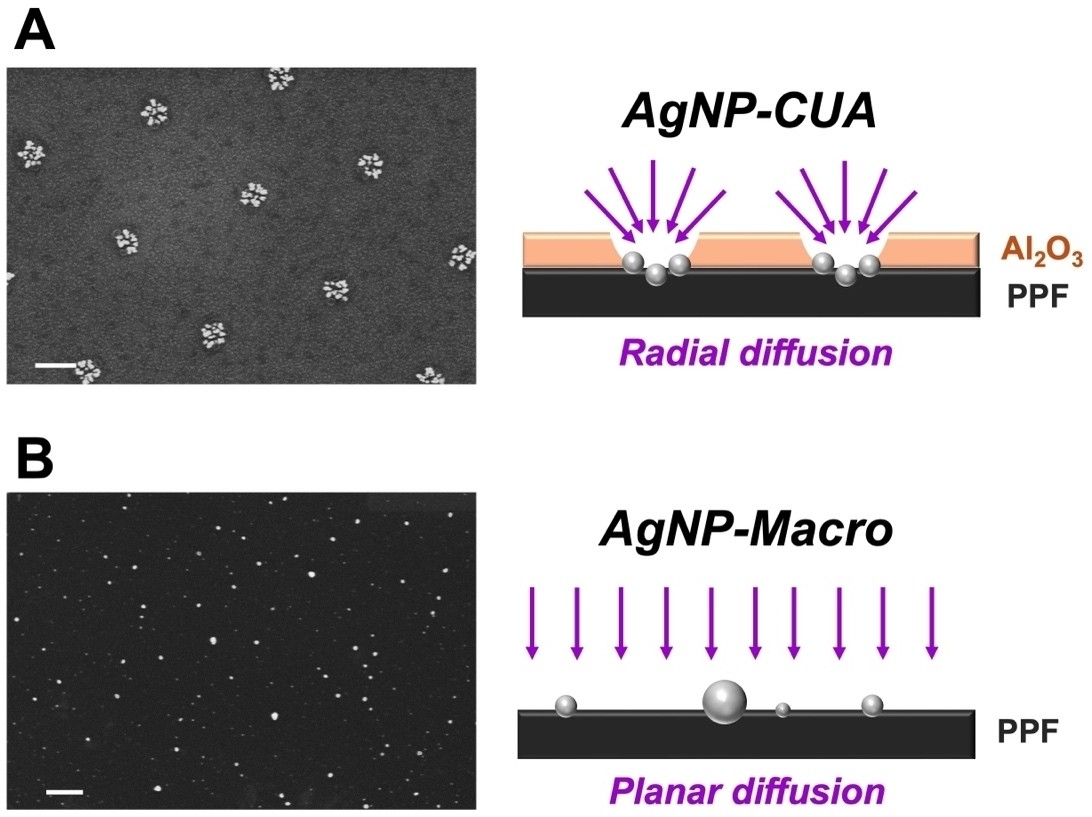
Representative scanning electron microscopy (SEM) images of silver nanoparticle (AgNP)‐modified (A) carbon ultramicroelectrode array (CUA) and (B) Macro electrode (left) and graphical representations of associated diffusion profiles on each electrode surface (right). Electrodeposition parameters were the same for both electrodes at 250 μM AgNO_3_ concentration, −0.6 V vs. SCE potential, and 60 s deposition time. The scale bars are 500 nm for both SEM micrographs.

The shorter deposition times and lower silver ion concentrations for achieving effective AgNP deposition in this study can be attributed to the unique physical properties and surface processes associated with the array‐based electrode geometry and nanometer‐sized electrodes (91±7 nm average radius) within the CUA design.^[46]^ Control studies were conducted using Macro electrodes (i. e., planar electrodes consisting of entirely PPF without the array of ultramicroelectrodes) to highlight the differences in AgNP electrodeposition. The distinct geometries and sizes of the planar macro‐sized electrode and the nanometer‐sized CUA electrode result in different diffusion profiles. The array‐based structure of the CUAs generates radial diffusion profiles,[^47^, ^64^, ^65^] characteristic of ultamicroelectrodes.^[47]^ Figure 4 presents SEM results from AgNP electrodeposition (at −0.6 V vs. SCE for 30 s using 250 μM AgNO_3_) on CUAs and Macro electrodes, along with a graphical representation of the distinct diffusion profiles. Due to the nanoscale dimensions of the individual electrodes within the CUA, electroactive species undergo hemispherical radial diffusion (Figure 4A), leading to increased mass transport rates and flux of species toward the electrode surface.[^47^, ^62^, ^63^, ^66^] In contrast, the planar Macro electrodes exhibit semi‐infinite linear diffusion, stemming from their inherently larger electrode area, which limits the mass transport rates and flux of species to the electrode surface.^[63]^ The differences in diffusion profile between the CUAs and planar Macro electrodes were evident in the cyclic voltammetric (CV) measurements performed using a purely reversible redox active species (Figure S5). Specifically, ferrocene methanol (FcMeOH) was employed as a standard redox probe. Figure S5A shows sigmoidal current‐potential profiles indicative of radial diffusion profiles on the CUAs, while the planar Macro electrode displays peak‐shaped CV traces (Figure S5B), characteristic of semi‐infinite linear diffusion processes.[^62^, ^63^] An additional control study was conducted at the same electrodeposition parameters (−0.6 V vs. SCE for 30 s in 250 AgNO_3_) on a substrate comprised of entirely aluminum oxide (Al_2_O_3_). The absence of AgNP deposition on the metal oxide Al_2_O_3_ layer (Figure S6) emphasizes the insulating nature of this material.

When considering diffusion mass transport processes, it's important to recognize the complexity of the CUA electrode platform. Diffusion over the CUAs can be classified into several types: (1) planar diffusion over individual ultramicroelectrodes, (2) a mixed diffusion layer over individual ultramicroelectrodes that transitions between planar and hemispherical diffusion, (3) hemispherical diffusion layers over individual ultramicroelectrodes, and (4) a mixed diffusion layer resulting from the overlap of individual diffusion layers.^[64]^ Consequently, studying mass transport in recessed, cylindrical ultramicroelectrodes within the CUA array necessitates the numerical simulation of diffusion processes and the development of precise simulation models, as seen in prior studies on microelectrode arrays.[^64^, ^67^, ^68^, ^69^] These intriguing aspects will be theoretically modeled and explored in future research. In summary, AgNP electrodeposition on CUAs adhered to parameter trends similar to those reported for planar electrodes in previous studies. However, due to the unique array‐based electrode design with nanoelectrode sizes and radial diffusion profiles, successful AgNP deposition on CUAs was achieved with shorter deposition times and lower silver ion concentrations compared to earlier research.

### Amount of Silver Electrodeposited on Silver Nanoparticle (AgNP)‐Modified Carbon Ultramicroelectrode Arrays (CUAs)

The amperometric data from the electrodeposition of AgNPs were used to determine the quantity of silver on AgNP‐modified CUAs and planar Macro electrodes. The amount of silver was calculated from replicates (*n*=3) of three AgNP‐modified electrode samples, namely (1) CUAs modified at −0.6 V vs. SCE, (2) CUAs modified at −0.3 V vs. SCE, and (3) planar Macro electrodes modified at −0.6 V vs. SCE. The deposition times and AgNO_3_ concentrations were consistent across all samples at 30 s and 100 μM, respectively. Figure S7 displays representative current‐time traces for each AgNP‐modified electrode. Faraday's law (Equation S1) was employed to estimate the total quantity of silver on each AgNP‐modified electrode surface from the total charge passed through the electrochemical system. The total charge value was determined by integrating the area under each deposition *i‐t* curve from 3–30 s. These results are summarized in Table 1, including average values of the total charge, the amount of silver, and the amount of silver per electrode area for each AgNP‐modified electrode sample.

**Table 1:** Values for the total charge, total amount of silver deposited, and moles of Ag per electrode area during electrodeposition at potentials of −0.6 V and −0.3 V vs. SCE for the silver nanoparticle modified carbon ultramicroelectrode arrays (AgNP‐CUAs) and −0.6 V for the AgNP‐Macro electrode. The deposition times and AgNO_3_ concentrations were the same for all electrodes at 30 s and 100 μM, respectively. Results are reported as averages from *n*=3 replicates.

Electrode	Deposition Potential (V vs. SCE)	Charge (μC)	Amount of Silver (nmol)	Amount of Silver per Electrode Area (nmol cm^−2^)
AgNP‐CUA	−0.6	800±200	8±2	1100±200
AgNP‐CUA	−0.3	30±30	0.3±0.3	40±40
AgNP‐Macro	−0.6	1240±30	12.6±0.4	26.0±0.9

It is important to note that the total CUA electrode area is 0.0071 cm^2^, corresponding to 1.4 % total exposed carbon area, while the Macro‐sized PPF electrode, with 100 % exposed carbon, has an electrode area of 0.495 cm^2^.[^46^, ^47^] To account for the differences in electrode sizes, the moles of Ag determined on each AgNP‐functionalized electrode surface were normalized by the electrode area for both the CUAs and planar Macro electrodes. This normalization provides comparative data on the AgNP amounts electrodeposited on each electrode surface, independent of the electrode area size. The AgNP‐functionalized CUAs modified with the deposition potential of −0.6 V vs. SCE had the highest silver amount per electrode area, estimated to be 1100±200 nmol cm^−2^. In contrast, the Ag amount on the AgNP‐CUA electrode with the less reductive potential of −0.3 V vs. SCE was considerably lower, averaging 40±40 nmol cm^−2^. This data suggests that more reductive deposition potential values (i. e., higher electrochemical driving force) result in a greater quantity of silver on the CUA electrode surface. As a control, the amount of Ag per electrode area was also determined for the planar Macro electrodes modified with AgNPs at the more reductive electrodeposition potential of −0.6 V vs. SCE. The AgNP‐tailored planar Macro electrodes displayed the lowest amount of silver per area at 26.0±0.9 nmol cm^−2^, which is approximately 40 times less compared to the silver amount on the AgNP‐CUAs at this same deposition potential. Further research is required to mathematically establish the theoretical values of silver deposited on the recessed cylindrical ultramicroelectrodes within the CUA array as well as on the planar Macro electrodes. This will enable a comparison with the quantitative experimental values presented in this study. Overall, these results show the impact of electrode geometry and size, as well as applied electrochemical potential, on the amount of AgNPs deposited.

### Catalytic Enhancement of Hydrogen Peroxide Reduction Reaction from Silver Nanoparticle (AgNP)‐Modified Carbon Ultramicroelectrode Arrays (CUAs)

The enhanced electrocatalytic activity for the reduction of hydrogen peroxide (H_2_O_2_) on silver nanoparticle (AgNP)‐modified electrodes has been investigated previously for applications in electrochemical sensing and electrocatalysis.[^14^, ^29^] The standard reduction potential and proton‐coupled two‐electron electrochemical reduction reaction for H_2_O_2_ are shown in Equation (2). Various multi‐step reaction mechanisms have been proposed to understand the redox behavior of H_2_O_2_. Recent reports suggest that the mechanism involves the initial chemical degradation of H_2_O_2_ into water and oxygen, followed by the electrochemical reduction of oxygen.^[14]^ These mechanisms have been simulated and experimentally characterized in previous studies by the Compton Research Group.[^14^, ^29^] While a detailed discussion of the H₂O₂ reduction mechanism is beyond the scope of this paper, our study aims to demonstrate a proof‐of‐concept for enhancing the electrocatalytic response for H₂O₂ reduction using the AgNP‐modified CUA electrodes.

(2) $$H_{2}O_{2\left(l\right)}+\;{2e}^{-}+{2H}^{+}↔{2H}_{2}O_{\left(l\right)}\;\;E^{0\;}=1.763\;V\;vs.\;NHE$$

**Figure 5:**
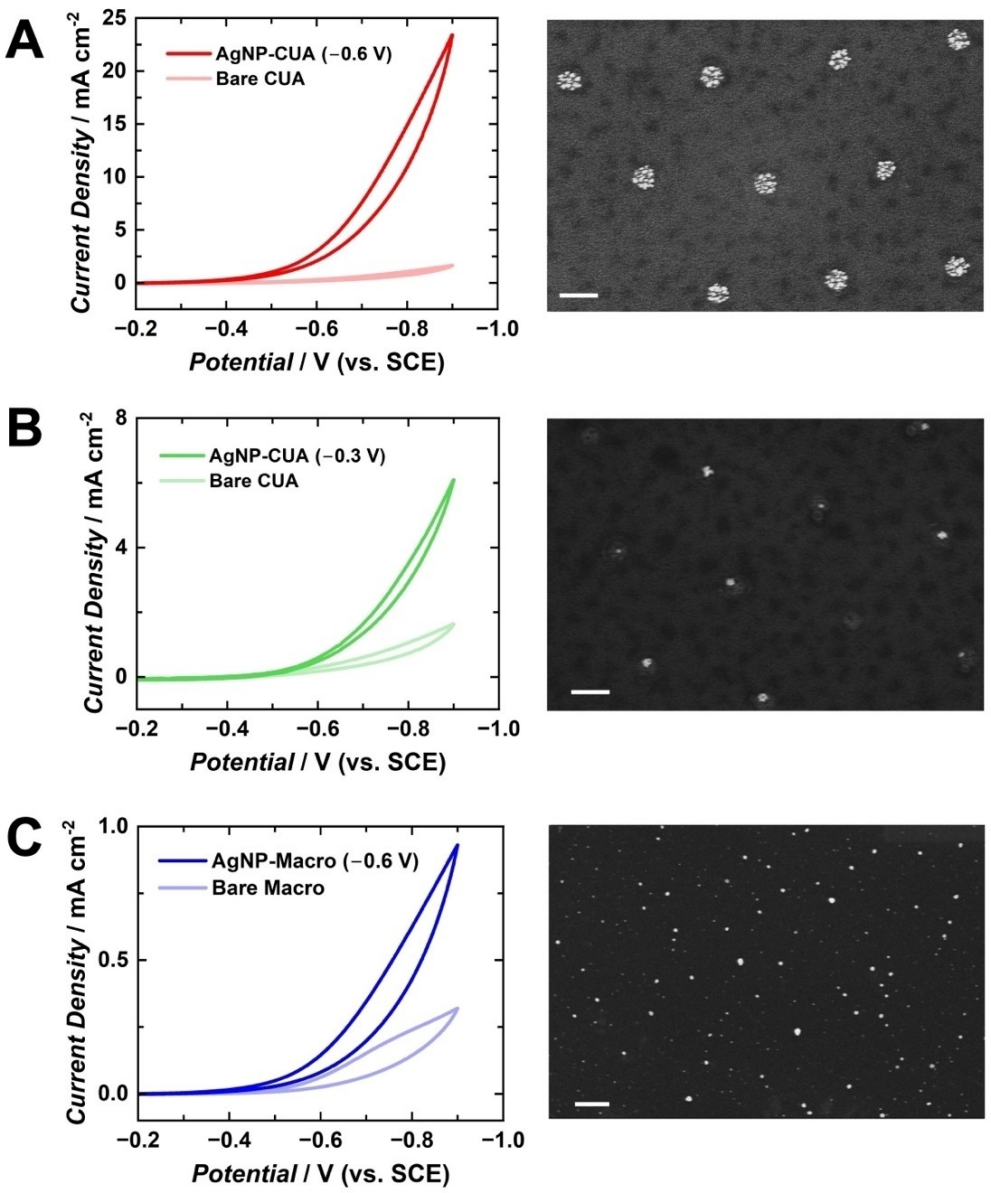
Cyclic voltammograms (CVs) of 10 mM H_2_O_2_ in 0.1 M sodium phosphate buffer (SPB), shown on the left, and scanning electron microscopy (SEM) images, displayed on the right, comparing carbon ultramicroelectrode arrays (CUAs) at deposition potentials of (A) −0.6 V and (B) −0.3 V vs. SCE, and (C) Macro electrodes at −0.6 V vs. SCE, depicted in red, green, and blue, respectively. The darker traces on each plot represent the silver nanoparticle (AgNP)‐modified electrodes (at deposition times and AgNO_3_ concentrations of 30 s and 100 μM, respectively) and the lighter trace indicates the current response on the bare, unmodified CUA or Macro electrode. All CVs were performed at scan rates of 50 mV s^−1^ and each current‐potential trace is reported as a representative replicate from *n*=3 individual measurements. The scale bars indicate 500 nm in all SEM micrographs.

**Figure 6:**
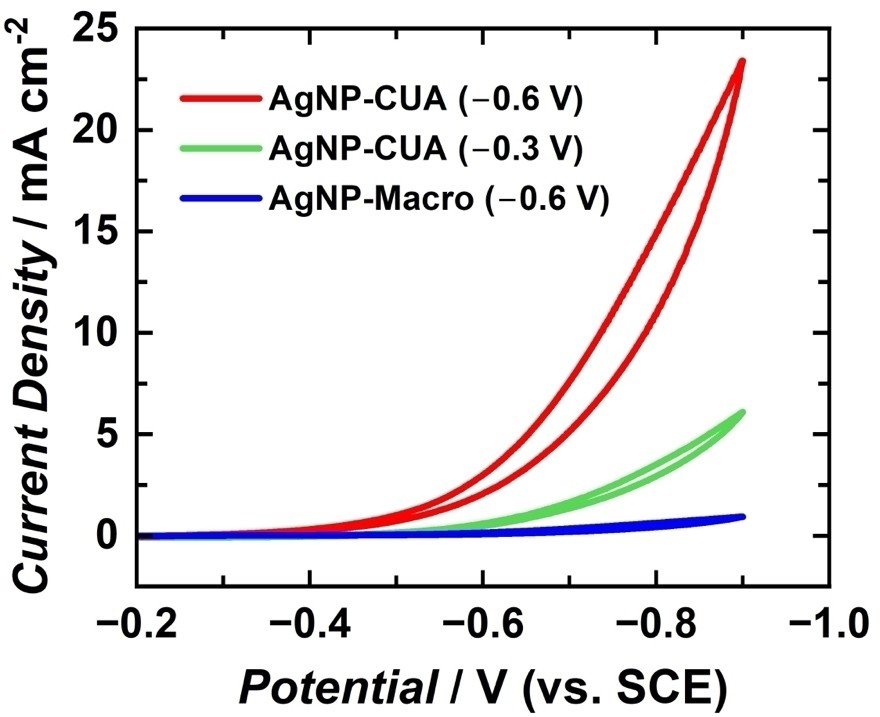
Cyclic voltammograms (CVs) of 10 mM H_2_O_2_ in 0.1 M sodium phosphate buffer (SPB) at a scan rate of 50 mV s^−1^ comparing carbon ultramicroelectrode arrays (CUAs) at deposition potentials of (A) −0.6 V and (B) −0.3 V vs. SCE, and (C) Macro electrodes at −0.6 V vs. SCE, depicted in red, green, and blue, respectively. Deposition times and AgNO_3_ concentrations for all CV traces were 30 s and 100 μM, respectively. Each CV current‐potential profiles are reported as a representative replicate from *n*=3 tests.

The electrocatalytic activity of AgNP‐modified electrodes is influenced by nanoparticle size, morphology, and count, all of which are directly affected by electrodeposition parameters, such as potential, time, and silver ion concentration.^[11]^ Cyclic voltammetry was employed to assess the impact of these parameters and electrode geometry on the electrocatalytic response of H_2_O_2_ reduction at AgNP‐modified electrodes. The H_2_O_2_ reduction reaction was studied at AgNP‐modified electrodes, including AgNP‐CUAs modified at deposition potentials of −0.6 V and −0.3 V vs. SCE, and planar Macro electrodes at −0.6 V vs. SCE. AgNPs were deposited at consistent times and Ag^+^ ion concentrations of 30 s and 100 μM, respectively, for all electrodes. In addition to the AgNP‐modified samples, control studies of H_2_O_2_ reduction were performed with bare, unmodified CUA and planar Macro electrodes. Voltammetric data from the electrochemical reduction of 10 mM H_2_O_2_ in buffered solution on AgNP‐modified and bare electrodes are displayed in Figures 5 and 6. Figure 5 includes SEM images of the AgNP‐modified electrode surfaces. The cathodic catalytic current values at −0.9 V vs. SCE corresponding to H_2_O_2_ reduction were normalized to the exposed carbon electrode area for CUAs and planar Macro electrodes, which are 0.0071 cm^2^ and 0.495 cm^2^, respectively. This normalization provided catalytic current densities, enabling comparison of electrocatalytic activity for H_2_O_2_ reduction independent of the electrode area size.

Table 2 summarizes the experimentally determined H_2_O_2_ cathodic current densities for each electrode sample. The AgNP‐CUAs, modified under more favorable deposition conditions (−0.6 V vs. SCE), exhibited an average H_2_O_2_ catalytic current density of 19±4 mA cm^−2^, which is an order of magnitude higher than the current density of the bare, unmodified CUA (1.9±0.5 mA cm^−2^). These results suggest that more reductive deposition potentials are associated with increased electrical current response, indicating enhanced electrocatalytic activity for the H_2_O_2_ reduction. This increase is likely due to the higher quantity of Ag on the electrode surface (Table 1) and the larger surface area of the AgNPs. Conversely, the AgNP‐CUAs modified at less reductive deposition potential conditions (−0.3 V vs. SCE) showed a current density value less than half that observed under more reductive conditions (−0.6 V vs. SCE), averaging 7±3 mA cm^−2^. The average H_2_O_2_ cathodic current density for the Macro electrodes was 0.99±0.06 mA cm^−2^, indicating marginal AgNP electrocatalytic activity for H_2_O_2_ reduction compared to the AgNP‐modified CUA samples. These findings are consistent with the quantities of silver deposited on the surfaces of the CUA and planar Macro electrodes, as detailed in Table 1. This study underscores the importance of establishing AgNP electrodeposition parameters for novel electrode platforms (e. g., CUAs) to achieve optimal Ag formation density for electrocatalysis applications.

**Table 2:** Cathodic current density values (determined at −0.9 V vs. SCE) for H_2_O_2_ reduction on silver nanoparticle (AgNP)‐modified carbon ultramicroelectrode arrays (CUAs) versus AgNP‐modified Macro electrodes. AgNPs were deposited at −0.6 V or a −0.3 V vs. SCE for 30 s in 100 μM of AgNO_3_. Current densities for the non‐modified, bare electrodes are also reported for comparison purposes. Values are reported as averages of *n*=3 replicate measurements.

Electrode	Deposition Potential (V vs. SCE)	H_2_O_2_ Cathodic Current Density (mA cm^−2^)
Bare CUA	N/A	1.9±0.5
Bare Macro	N/A	0.5±0.1
AgNP‐CUA	−0.6	19±4
AgNP‐CUA	−0.3	7±3
AgNP‐Macro	−0.6	0.99±0.06

## Conclusions

In summary, we investigated the impact of electrodeposition parameters—deposition potential, deposition time, and Ag^+^ concentration—on the formation of silver nanoparticles (AgNPs) on carbon ultramicroelectrode arrays (CUAs). Extensive scanning electrochemical microscopy analysis revealed trends in AgNP sizes and distribution across various experimental deposition potentials, times, and Ag^+^ concentrations. Our results indicated that more reductive deposition potentials (i. e., more negative potentials) were associated with higher counts of smaller AgNPs. Additionally, longer deposition times and higher Ag^+^ concentrations consistently produced larger AgNPs. The nano‐sized individual electrodes organized in the unique array‐based geometry of the CUA necessitated shorter deposition times (15–30 s) and lower Ag^+^ concentrations (50–100 μM) compared to previous studies with planar macrometer‐size electrodes, which used longer deposition periods and millimolar metal ion concentrations. The amount of silver deposited per electrode area was calculated for AgNP‐CUAs at deposition potentials of −0.6 V and −0.3 V vs. SCE, as well as for AgNP‐modified planar Macro electrodes at the more reductive deposition potential. The highest amount of silver per electrode area was observed on the AgNP‐CUAs modified at −0.6 V vs. SCE, with 1100±200 nmol cm^−2^, which was over 30 times greater than that on other AgNP‐modified electrode samples. To demonstrate the electrocatalytic activity of the AgNP‐modified electrodes, a voltammetric study of the H_2_O_2_ reduction reaction was performed with the same AgNP‐electrode samples from the silver quantity study. The AgNP‐CUAs modified at the more reductive deposition potential of −0.6 V vs. SCE exhibited the highest H_2_O_2_ cathodic current density at 19±4 mA cm^−2^, reflecting trends similar to those observed in silver deposition. Overall, our findings highlight the importance of evaluating electrodeposition parameters for AgNP formation on novel electrode surfaces, such as CUAs. The CUA design, characterized by array‐based geometry and size of the ultramicroelectrodes, along with experimental parameters (e. g., potential, time, and metal ion concentration), significantly influences the AgNP electrodeposition and electrocatalytic activity. Achieving a successful nanoparticle deposition with lower metal ion solution concentrations and shorter deposition periods, while still ensuring adequate particle formation on our array‐based CUA electrode platform, represents a significant advancement for the electrochemistry community.

## Author Contributions

O.S. – Conceptualization, Funding acquisition, Project administration, Resources, Supervision, Formal analysis, Investigation, Methodology, Writing‐original draft, Writing‐review & editing. C.J.W. – Formal analysis, Investigation, Methodology, Writing‐original draft. N.E.S. – Investigation, Writing–review & editing. E.M.V. – Investigation, Writing‐review & editing. All authors contributed to preparation and editing and have given approval to the final version of the manuscript.

## Conflict of Interests

The authors declare no conflict of interests.

1

## Supporting information

As a service to our authors and readers, this journal provides supporting information supplied by the authors. Such materials are peer reviewed and may be re‐organized for online delivery, but are not copy‐edited or typeset. Technical support issues arising from supporting information (other than missing files) should be addressed to the authors.

Supporting Information

## Data Availability

The data that support the findings of this study are available in the supplementary material of this article.
