# Supplementary material for: Electrochemical Deposition of Silver Nanoparticle Assemblies on Carbon Ultramicroelectrode Arrays
Source: Chemphyschem. 2025 Jan 8;26(5):e202400791. doi: 10.1002/cphc.202400791 (PMC11878751; doi:10.1002/cphc.202400791)
Supplement: Supplementary file 1 — Supporting Information [file CPHC-26-e202400791-s001.pdf]

ChemPhysChem

Supporting Information

Electrochemical Deposition of Silver Nanoparticle Assemblies on Carbon Ultramicroelectrode Arrays

Courtney J. Weber, Natalie E. Strom, Emma M. Vagnoni, and Olja Simoska*

ChemPhysChem

Supporting Information

Electrochemical Deposition of Silver Nanoparticle Assemblies on Carbon Ultramicroelectrode Arrays

Courtney J. Weber,[1] Natalie E. Strom,[1] Emma M. Vagnoni,[1] and Olja Simoska[1,*]

[1]*Department of Chemistry and Biochemistry, University of South Carolina, 631 Sumter Street, Columbia, SC 29208*

*Corresponding author: Dr. Olja Simoska (E-mail: osimoska@mailbox.sc.edu)

Contents **Page No.**

Cyclic voltammograms of $AgNO_3$ concentration-dependent and scan-dependent studies	....	S3
EDX results of AgNPs on a single electrode in the CUA from SEM images	....	S4
Particle size histograms and respective SEM images of AgNP-modified CUAs for 50 μM of $AgNO_3$	....	S5
Particle size histograms and respective SEM images of AgNP-modified CUAs for 250 μM of $AgNO_3$	....	S6
Cyclic voltammograms of ferrocenemethanol on the CUA and Macro electrodes	....	S7
Qualitative SEM comparison of AgNP electrodeposition on the CUA, Macro, and Al_2O_3 surfaces	....	S8
Representative amperometric *i-t* traces for the electrodeposition of AgNP on CUAs and Macro electrodes	....	S9
Calculation and determination method for AgNP amount deposited	....	S10
References	....	S11

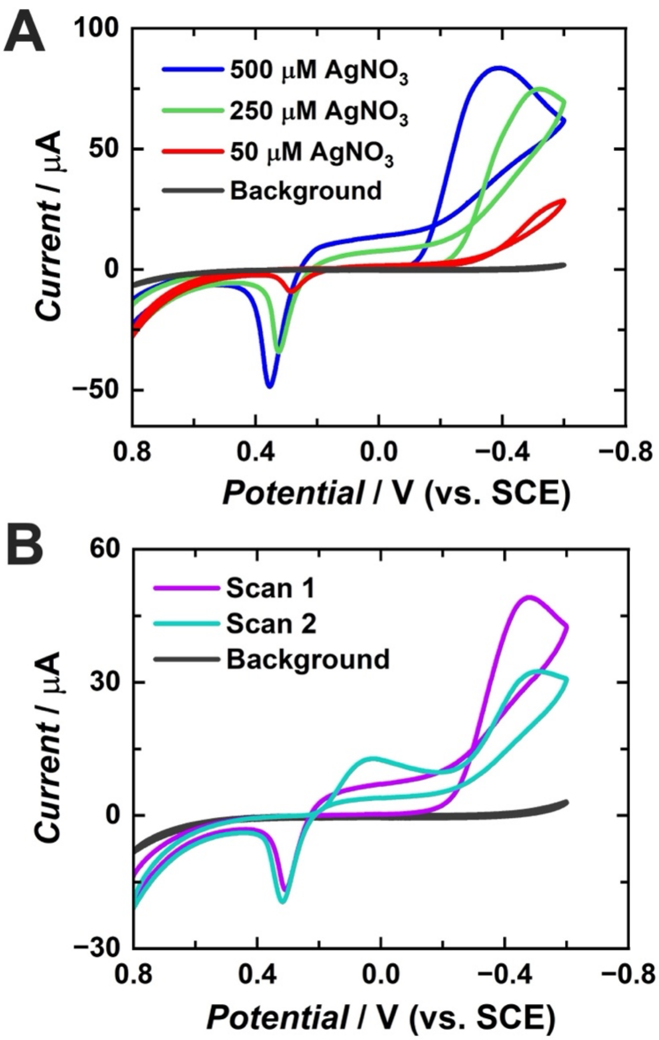

Figure S1. Cyclic voltammetric (CV) profiles of (A) concentration-dependent studies with 50 μM (red trace), 100 μM (green curve), and 250 μM (blue trace) of $AgNO_3$ solution and (B) scan-dependent studies at a scan rate of 100 mV s^{-1}. Scan two was performed immediately after scan one for the scan-dependent study in a solution of 250 μM $AgNO_3$. All solutions included 0.5 M KNO_3 as the supporting electrolyte.

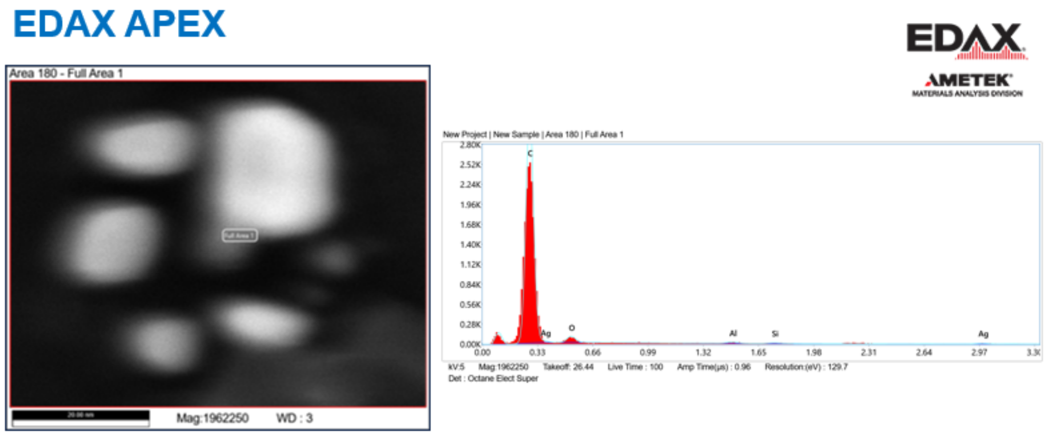

eZAF Quant Result - Analysis Uncertainty: 14.13 %

Element	Weight %	Atomic %	Error %	Net Int.	R	A	F
C K	80.1	94.0	6.4	175.3	0.9260	0.7992	1.0000
O K	3.6	3.2	14.8	5.3	0.9390	0.5191	1.0000
Al K	1.1	0.6	28.8	1.5	0.9637	0.9486	1.0065
Si K	0.7	0.4	61.4	0.8	0.9682	0.9664	1.0093
Ag L	14.5	1.9	53.2	1.0	0.9895	0.9954	1.0044

Figure S2. Energy dispersive x-ray (EDX) spectroscopic measurements were performed on silver nanoparticles (AgNPs) within an individual electrode on the carbon ultramicroelectrode array (CUA) surface. This was achieved via scanning electron microscopy (SEM) imaging to confirm the presence of silver on the electrode surface. In addition to Ag, other detected elements included carbon (C) from the electrode material, oxygen and aluminum (O and Al) from the alumina layer, and silicon and oxygen (Si and O) from the quartz substrate.

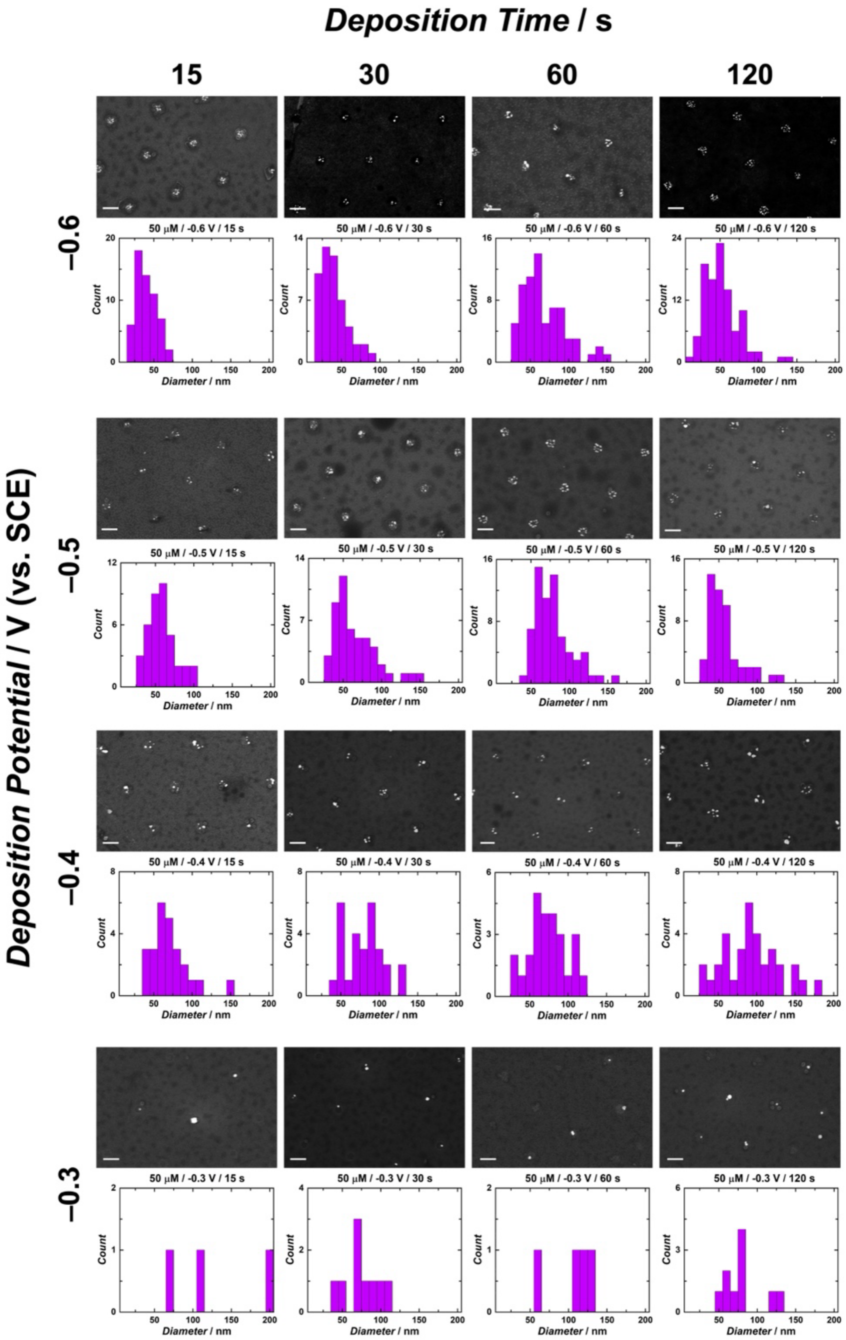

Figure S3. Silver nanoparticle (AgNP) size distribution histograms displayed with representative scanning electron microscopy (SEM) images for each electrodeposition potential and time parameter*s* using 50 µM $AgNO_3$ in 0.5 M KNO_3 supporting electrolyte solution on carbon ultramicroelectrode arrays (CUAs). The data is organized according to deposition time (increasing from left to right) and potential (less reductive, or more positive, from top to bottom). Measurements for particle diameters, as displayed in the histograms, were recorded from n=9 individual electrodes of the same area on one CUA surface. The scale bars in each SEM micrograph are 500 nm in length.

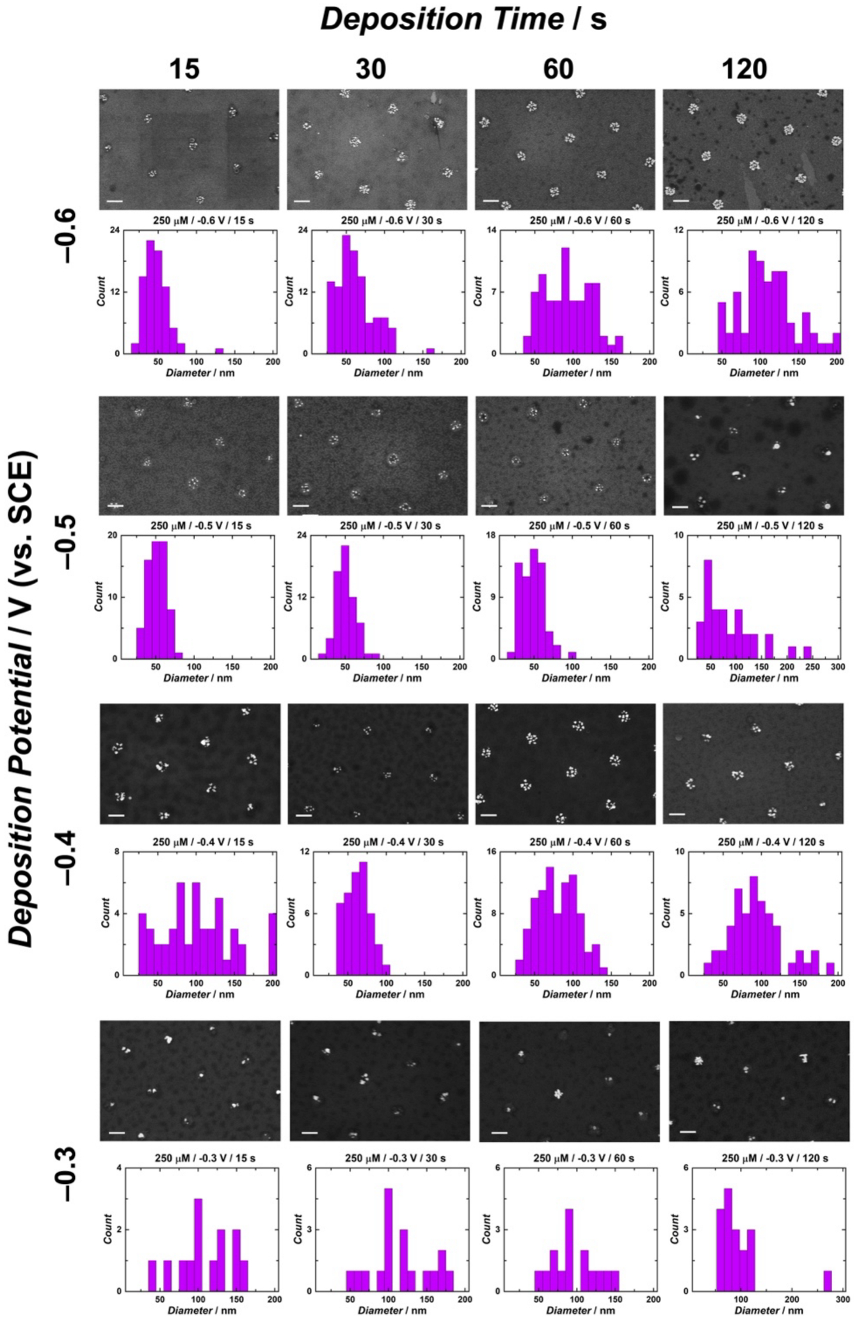

Figure S4. Silver nanoparticle (AgNP) size distribution histograms displayed with representative scanning electron microscopy (SEM) images for each electrodeposition potential and time parameters using 250 μM $AgNO_3$ in 0.5 M KNO_3 supporting electrolyte solution on carbon ultramicroelectrode arrays (CUAs). The data is organized according to deposition time (increasing from left to right) and potential (less reductive, or more positive, from top to bottom). Measurements for particle diameters, as displayed in the histograms, were recorded from n=9 individual electrodes of the same area on one CUA surface. The scale bars in each SEM micrograph are 500 nm in length.

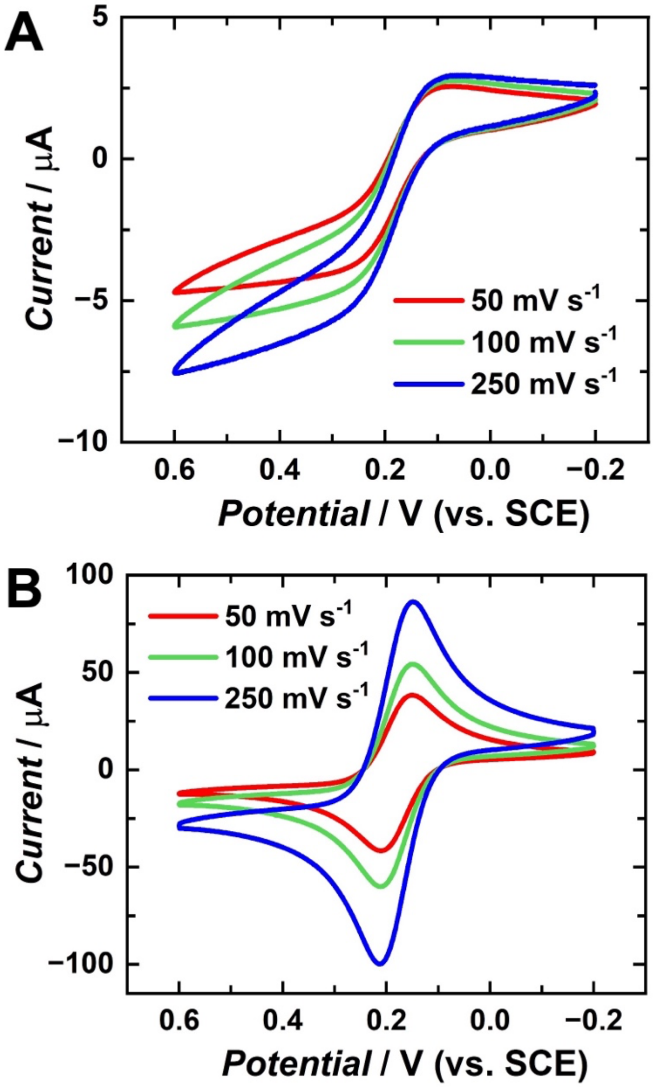

Figure S5. Cyclic voltammograms of 500 μM ferrocenemethanol in 0.5 M KCl solution at scan rates of 50, 100, and 250 mV s^{-1}. Voltammetric studies were performed on (A) carbon ultramicroelectrode arrays (CUAs) and (B) Macro electrodes to compare the diffusion mass transport profiles on each electrode.

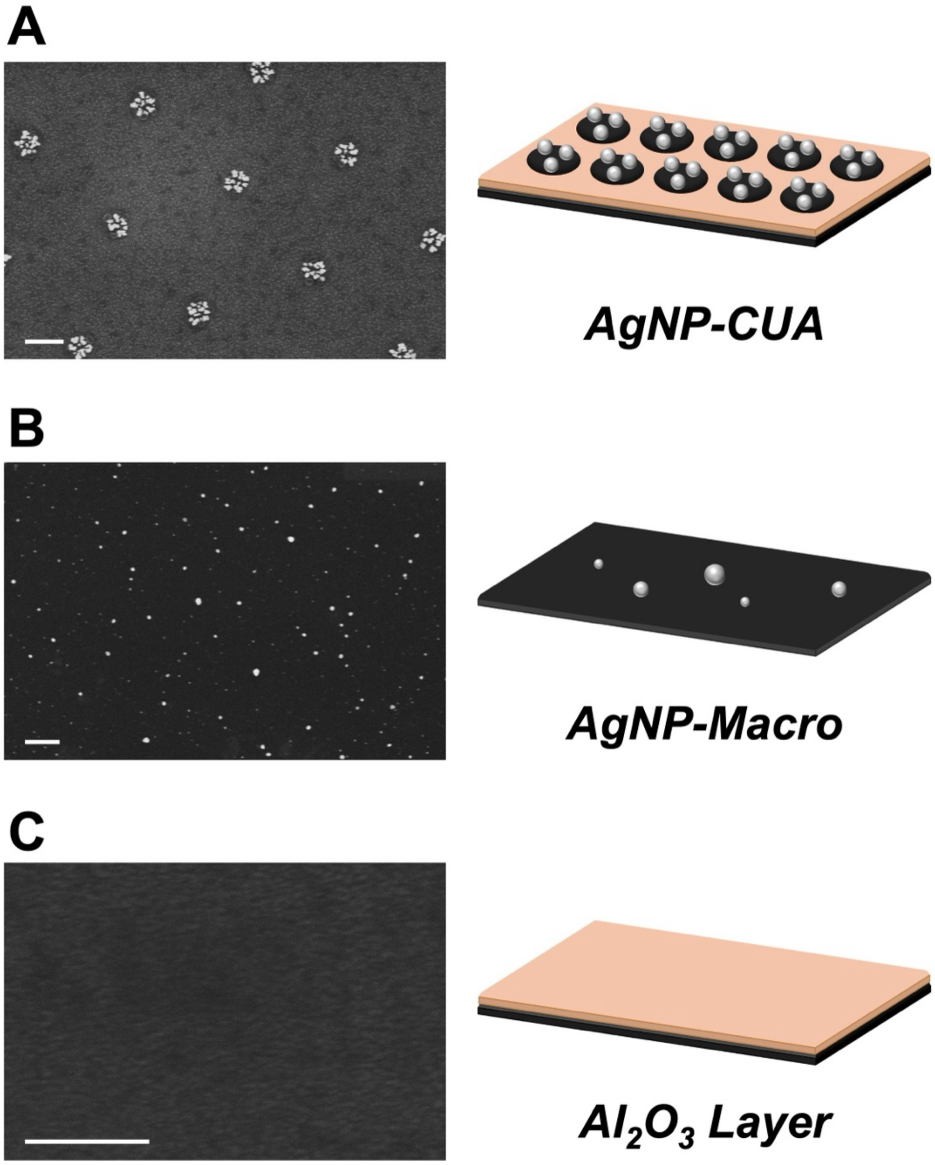

Figure S6. Representative scanning electron microscopy (SEM) images and graphical representations of the electrochemical deposition of silver nanoparticles (AgNPs) on (A) carbon ultramicroelectrode arrays (AgNP-CUAs), (B) Macro carbon electrodes (AgNP-Macro), and (C) aluminum oxide (Al_2O_3) surfaces. The insulating Al_2O_3 surface functions as a control sample due to evaluate AgNP electrodeposition on the 10 nm Al_2O_3 layer utilized in CUA the design. AgNPs were deposited at –0.6 V vs. SCE for 60 s in a solution of 250 μM $AgNO_3$ and 0.5 M KNO_3 for all three surfaces. The scale bars are 500 nm in length.

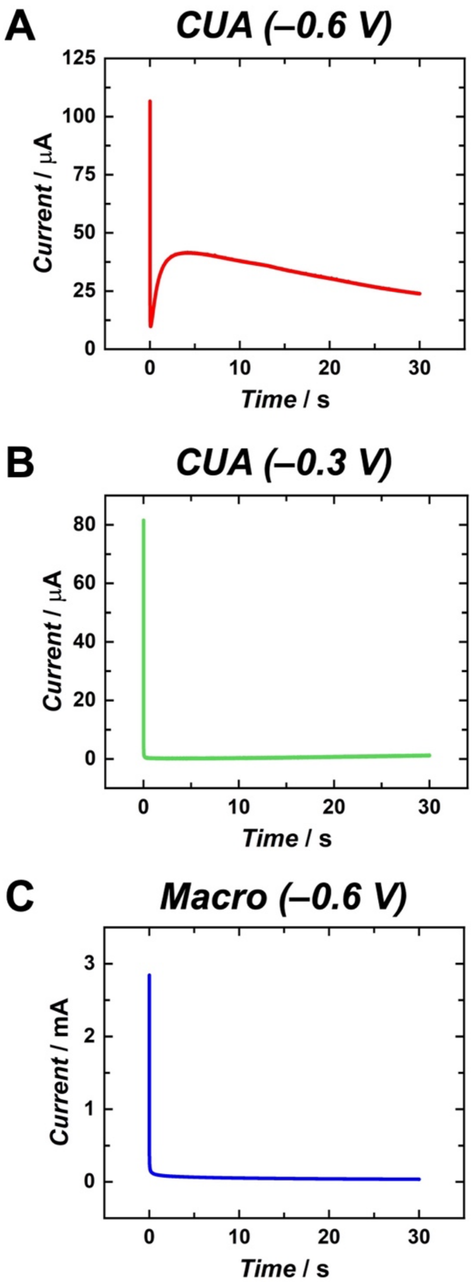

Figure S7. Representative amperometric current-time (*i*-*t*) transients for the electrodeposition of silver nanoparticles (AgNPs) on (A–B) carbon ultramicroelectrode arrays (CUAs) and (C) Macro electrodes. AgNPs were deposited at (A, C) –0.6 V or (B) –0.3 V vs. SCE for 30 s in a solution containing 100 μM $AgNO_3$ and 0.5 M KNO_3.

Determination of Silver Nanoparticle (AgNP) Quantity Deposited on CUAs

The quantity of silver on the CUA surfaces was determined via Faraday's law calculation.[1] Faraday's law allows for the determination of chemical substance amount (in moles) from charge (in coulombs), as displayed in **Equation S1**:

$$\boldsymbol{Q = nNF} \qquad \textbf{Equation (S1)}$$

For this equation, Q indicates the total charge passed through the electrochemical system in Coulombs (C), n represents the number of electrons transferred in the redox reaction of interest, N denotes the species amount in moles (mol), and F symbolizes Faraday's constant (96,485 C mol^{-1}). The total charge value was experimentally determined by integrating the area under the amperometric trace (**Figure S7**) from 3–30 s. The first three seconds (or 10%) of data points in the *i-t* curve were excluded from the charge calculation due to non-faradaic current contributions that occur at the beginning of the amperometric measurement.[2] Lastly, considering the redox reaction of Ag^+ to solid Ag^0 (**Equation 1**), the number of electrons (n) is equal to 1.

References

[1] A. J. Bard, L. R. Faulkner, H. S. White, *Electrochemical methods: fundamentals and applications*, 3rd ed., John Wiley & Sons, Inc., **2022**.

[2] O. Simoska, S. D. Minteer, *Techniques in electroanalytical chemistry*, American Chemical Society, ACS In Focus E-book Series, **2022**.
